# Evaluation of Better Staining Method among Hematoxylin and Eosin, Giemsa and Periodic Acid Schiff-Alcian Blue for the Detection of Helicobacter pylori in Gastric Biopsies

**DOI:** 10.21315/mjms2020.27.5.6

**Published:** 2020-10-27

**Authors:** Abdullah Saleh Alkhamiss

**Affiliations:** Department of Pathology and Laboratory Medicine, Collage of Medicine, Qassim University, Qassim, Saudi Arabia

**Keywords:** gastric biopsies, gastritis, Helicobacter pylori, intestinal metaplasia, Giemsa, periodic acid Schiff-Alcian blue

## Abstract

**Background:**

This study was undertaken to evaluate the preferred method (Giemsa or periodic acid Schiff-Alcian blue [PAS-AB] stains) of detecting *Helicobacter pylori* (*H. pylori*) in gastric mucosal biopsies in terms of sensitivity, specificity and applicability. To the best of my knowledge, this is the first report comparing Giemsa and PAS-AB staining for the detection of *H. pylori* in such biopsies.

**Methods:**

The formalin-fixed paraffin-embedded blocks of 49 gastric biopsies from different patients were collected from the archive of anatomical pathology at King Abdulaziz Medical City, National Guard, Riyadh, Saudi Arabia. From each block, three slides were prepared and analysed using the hematoxylin and eosin (H&E), Giemsa and PAS-AB stains to detect the presence/absence of *H. pylori*, and the results were compared in terms of sensitivity, specificity and applicability.

**Results:**

The majority of the biopsies in this study showed antrum-type gastric mucosa. Only 15 biopsies showed active gastritis, whereas the rest showed chronic gastritis. Three biopsies showed intestinal metaplasia. All were detected by PAS-AB stain, but only two-thirds were detected by H&E stain. Fifteen gastric biopsies showed *H. pylori* infection in general and in 13 of them, active gastritis cases were discovered. Fourteen out of these 15 *H. pylori* infection cases were detected by Giemsa stain, whereas only 13 cases were detected by H&E stain. PAS-AB stain showed the worst results since it demonstrated only 40% sensitivity and 67.65% specificity in *H. pylori* detection.

**Conclusion:**

Giemsa stain has better sensitivity and specificity in gastric *H. pylori* infection detection than PAS-AB. Therefore, using PAS-AB stain to detect *H. pylori* infection is not recommended.

## Introduction

*Helicobacter pylori* (*H. pylori*) is a well-defined, spiral-shaped, gram-negative bacterium responsible for the onset of several gastric pathologies ranging from mild gastritis to gastric malignancies (1). This microorganism was first discovered and described in a 1985 article by Marshall et al. (2). Since then, many studies have been conducted to explore this kind of bacterium and to highlight more about its significance in gastric pathology (3–8). At the same time, other researchers have tried to determine the most suitable method of diagnosing this infection in order to treat patients and protect them from serious complications (3).

Two broad methods are used to detect *H. pylori* in routine clinical diagnosis: invasive and non-invasive methods. In invasive methods, an endoscopic gastric biopsy is taken to be examined and tested histologically and cultured, with special stains, immune stains, polymerase chain reaction (PCR) tests and a rapid urase test (*Campylobacter*-like organism [CLO] test). The non-invasive methods include many tests such as the urea breath test (UBT), serological tests and detection of the *H. pylori* antigen in urine, blood and stool samples (3, 9–13). Each method has its own advantages and disadvantages. Detection of *H. pylori* in gastric biopsies is the gold standard method of detecting *H. pylori* infection, but exactly what should be used as a panel of tests in these biopsies remains controversial (1, 9, 11–14).

Several histopathological staining panels have been used for many years to detect *H. pylori* in gastric biopsies, including the combination of the hematoxylin and eosin (H&E) stain and other special stains, such as the Giemsa, periodic acid Schiff-Alcian blue (PAS-AB), methylene blue and Warthin-Starry silver stains (3, 13, 15). Other detection methods, such as immunohistochemistry (IHC) and fluorescent in-situ hybridisation-based staining methods are also available for *H. pylori* detection (14, 16–18). Molecular biology techniques, such as PCR tests, are also used to diagnosis *H. pylori* infection (13, 19).

It is now well documented that chronic gastritis-related disorders are one of the most prevalent causes of death in humans and their incidence rate is continuously on the rise globally, including in Saudi Arabia (20–21). Despite this, the prognosis for advanced gastric disorders is still limited and poor. Therefore, early detection of these disorders is extremely important for better treatment of patients with gastric disorders (1). The Giemsa and PAS-AB stains are the most common panels used routinely in gastric biopsies. The former is used to detect *H. pylori* infection, whereas the latter is used to highlight the presence of intestinal metaplasia in these biopsies.

The role of PAS-AB stain (compared to Giemsa stain) in the detection of *H. pylori* still needs further exploration, as some pathologists claimed that Giemsa is a better stain in *H. pylori* detection compared to other stains. Others claimed that the PAS-AB stain is as good for this purpose as the Giemsa stain. Hence, the selected panel of staining for detecting *H. pylori* in gastric biopsies appears controversial and confusing. Therefore, this study is designed to investigate the detection methods and to determine the preferred method among Giemsa and PAS-AB stains in detecting *H. pylori* in gastric biopsies. The specific aim is to compare the sensitivity, specificity and applicability of Giemsa and PAS-AB stains in detecting *H. pylori* in gastric biopsies.

## Methods

After receiving ethical approval from the regional ethical committee as well as obtaining the patients’ informed written consents, 56 formalin-fixed paraffin-embedded blocks from gastric biopsies and their glass slides from different patients were collected. These represented all the gastric biopsy cases from 15 May 2018–31 May 2018 in the archive of the Anatomical Pathology Department at King Abdulaziz Medical City, Ministry of National Guard, Riyadh City, Saudi Arabia. From each block, three glass slides were prepared and stained with H&E, Giemsa and PAS-AB stains, according to the standard protocols. Each glass slide was examined blindly by a pathologist for the presence/absence of *H. pylori*, without knowing the results from other stains of the same biopsy. At the same time, two other pathologists examined the slides in combination, to be controls for the study. That is, they looked at the H&E, Giemsa and PAS-AB glass slides together for each case, to determine the presence/absence of the *H. pylori*.

The main inclusion criterion was the presence of gastritis and the primary exclusion criterion included cases with dysplasia or gastric carcinomas, since the presence of these kinds of lesions could be indirect hints of the presence of *H. pylori* infection in the biopsy. Therefore, 7 cases were excluded and we finished with only 49 paraffin blocks.

## Results

A total of 49 patients participated in this study, ranging in age from 22–63 years. Eighteen patients were male and 31 patients were female. The majority of the histological types of the biopsies showed antrum-type gastric mucosa (Table 1).

**Figure 1:**
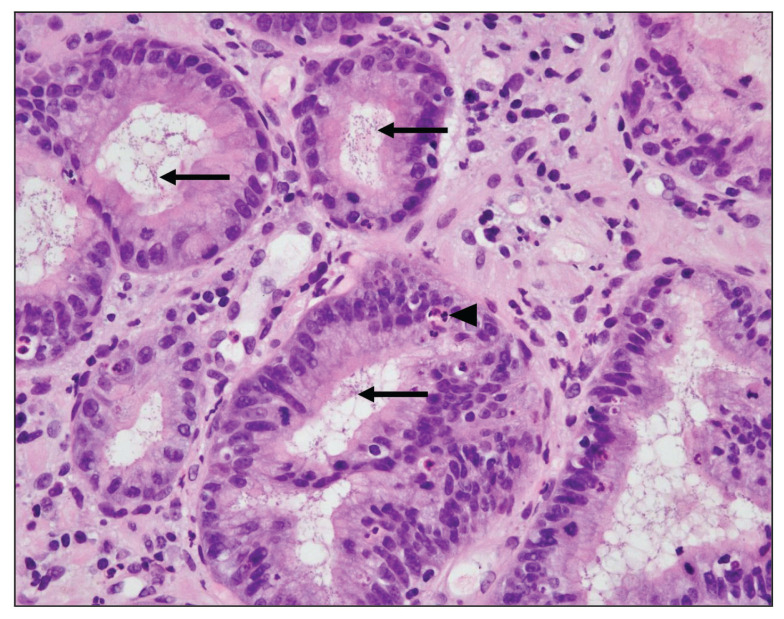
Gastric inflammation: Chronic active gastritis (◂) with many *H. pylori* microorganisms (⬅) in the glands (H&E stain 400×).

**Figure 2:**
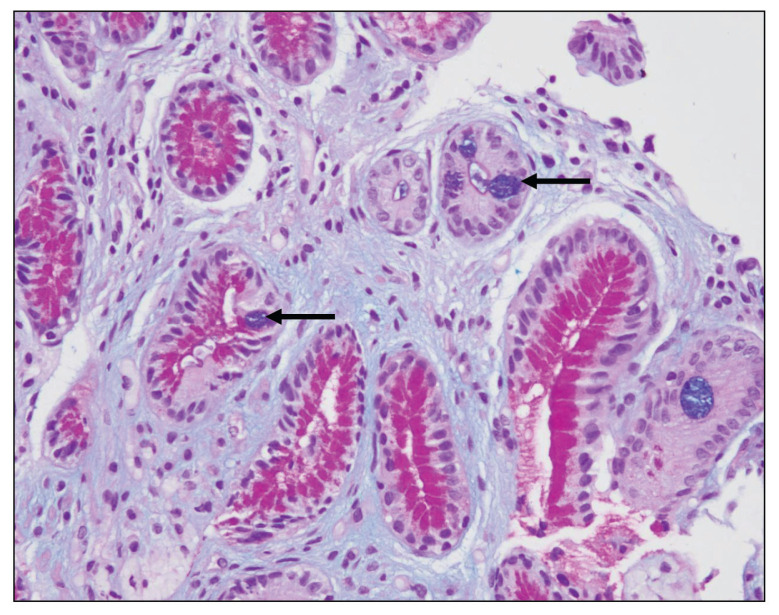
Intestinal metaplasia in the stomach: A small focus of intestinal metaplasia that was missed by the H&E stain and detected by PAS-AB stain, which highlights the goblet cells (⬅) in blue colour due to its mucin nature (PAS-AB stain 400×)

**Figure 3:**
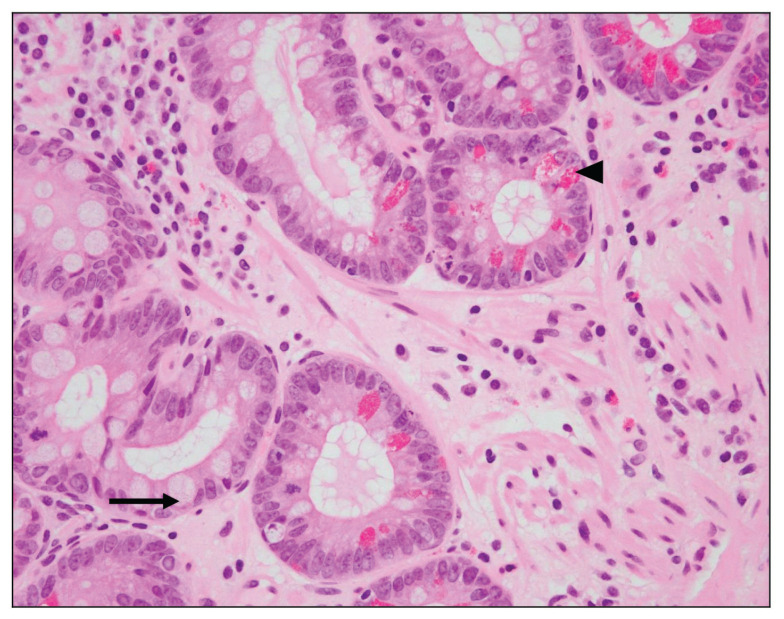
Gastric mucosa with intestinal metaplasia: There are many goblet cells (➞) in this view associated with Paneth cell metaplasia (◂) in the glands of the stomach (H&E stain 400×).

Fifteen biopsies showed chronic active gastritis (Figure 1), whereas the remaining 34 showed only chronic gastritis (Table 1). Three cases showed intestinal metaplasia (Table 2). All of these were detected by PAS-AB stain (Figure 2). In contrast, only two-thirds of them were detected by routine H&E stain (Figure 3). The reason was that the lesion was focal in the biopsy.

**Figure 4:**
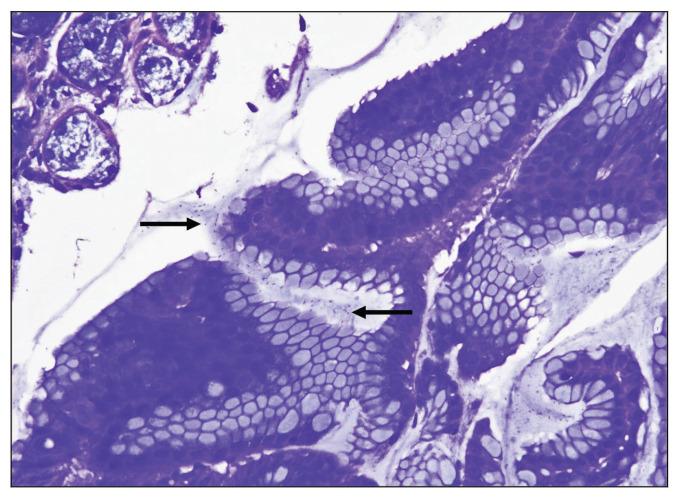
Detection of *H. pylori* gastritis by Giemsa stain: Many *H. pylori* microorganisms (➞) become clearly visible either inside the glands or on the surface of the mucosa (Giemsa stain 400×)

**Figure 5:**
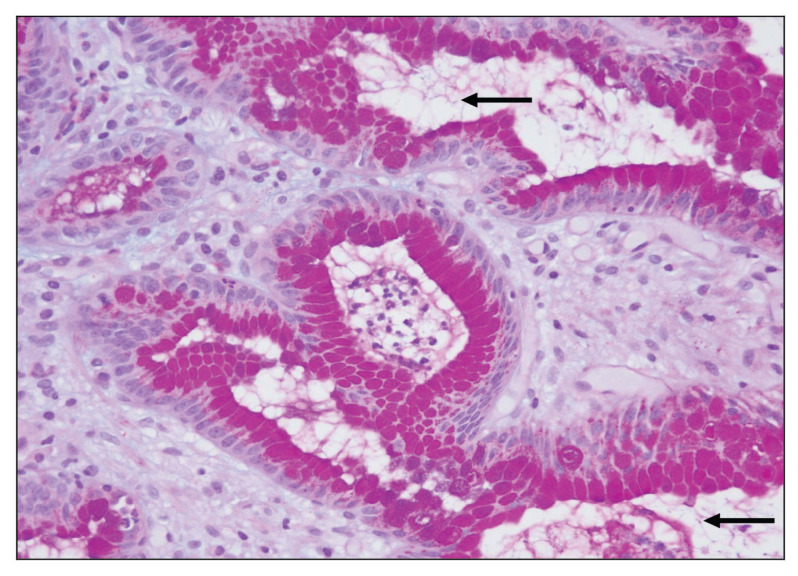
Detection of *H. pylori* gastritis by PAS-AB stain: The detection of *H. pylori* microorganisms (⬅) becomes very difficult due to the dirty background of the stain in comparison to Figures 1 and 4 which represent the same case (PAS-AB stain 400×)

**Figure 6:**
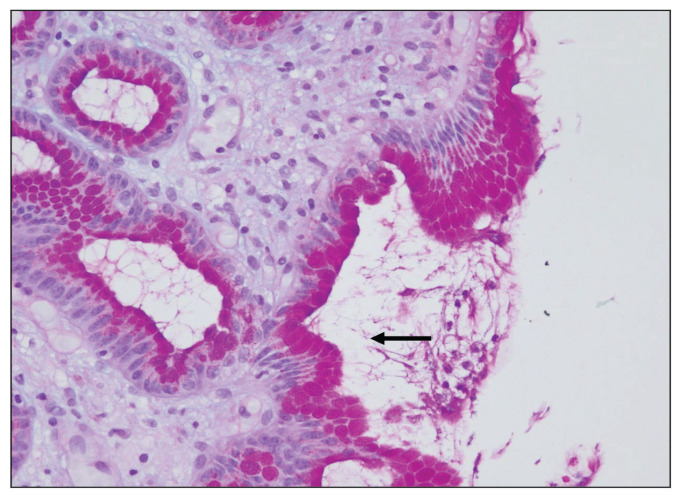
Gastritis without *H. pylori* infection: The background of the stain creates a delusion about the presence of fake *H. pylori* microorganisms (⬅). This case had been diagnosed as positive for the infection on PAS-AB, but when it compared with both H&E and Giemsa stains, it become clearly negative (PAS-AB stain 400×)

Fifteen gastric biopsies were found to be positive for *H. pylori* infection (through the combination of H&E stain, Giemsa stain, PAS-AB stain, the Urea breath test and the CLO test). These cases were distributed as follows: 13 cases showed chronic active gastritis and 2 cases showed only chronic gastritis. The sensitivity and specificity of H&E, PAS-AB and Giemsa stains in the detection of *H. pylori* are shown in Table 3. From this table, it is clear that Giemsa stain was superior to both H&E and PAS-AB stains in the detection of *H. pylori* with 93.33% sensitivity and 100% specificity (Figure 4). It is also clear that among the three stains, PAS-AB performed the worst since it had high false positivity as well as false negativity. This is mostly because of the PAS-AB dirty background of the slide, which made it difficult to differentiate between true *H. pylori* infection and the dirty background, as in Figures 5 and 6. In *H. pylori* detection, H&E stain had good specificity; however, its sensitivity was relatively low (Figure 1).

## Discussion

The detection of *H. pylori* infection through endoscopic features alone is not appropriate for a diagnosis. Therefore, these must be combined with the histopathological report (22, 23). This is necessary because: i) the *H. pylori* microorganism cannot be seen endoscopically; ii) there are many types of mucosal alteration that can be associated with *H. pylori* infection (such as gastritis, mucosal atrophy, ulceration, erosion, polyps and neoplasms) (24); and iii) there is no correlation between endoscopic findings and histopathological findings in patients with *H. pylori* infection (25).

Many researchers have concluded that the most common microscopic mucosal changes associated with *H. pylori* infection are active gastritis and/or the presence of lymphoid follicles with germinal center formation (16, 24–26). This is also supported by our research, since 86.67% of the *H. pylori* infection cases were associated with active gastritis. Yet if one considers the sensitivity, specificity, cost, reproducibility and rapidity of any test, there is no single gold standard test to detect *H. pylori* infection that has all these parameters (13). However, gastric biopsies are the gold standard method to detect this infection (1, 9, 11–14). The question is which panel to use in these biopsies. In addition to the standard H&E stain, many panels of stains have been proposed for this purpose, including Giemsa, PAS-AB, Warthin-Starry and IHC stains (13). However, some authors still suggest that ancillary studies should be done only for cases in which there is a high suspicion of the presence of *H. pylori* infection that cannot be visually seen by H&E (such as cases with active gastritis or with germinal centre formation) (13, 23, 28–32). The advantages of H&E alone are that: i) it is inexpensive; ii) it is a rapid test; and iii) it allows the evaluation of the presence of *H. pylori* as well as its complications (such as intestinal metaplasia and neoplasms) (13, 30, 32, 34). The disadvantages of this method are that: i) it will not detect *H. pylori* in cases with mild gastritis and a small quantity of bacteria; and ii) it overlooks this bacterium in patients who have been partially treated with proton pump inhibitors and/or antibiotics (13, 31–32). According to our research, the specificity of H&E in *H. pylori* detection is high (91.18%); however, its sensitivity is low (66.67%).

Giemsa stain is a simple, rapid and inexpensive stain that has good sensitivity, specificity and consistency in the detection of *H. pylori* infection (13, 27, 33, 34). Therefore, it is routinely used in some institutions. According to many scientific papers, Giemsa stain is superior to H&E in the detection of *H. pylori* (13, 27, 30, 33, 34). Based on our findings, the sensitivity of Giemsa stain in the detection of *H. pylori* is 93.33% while its specificity is 100%, which makes it better than H&E and PAS-AB stains.

The data from this study showed that PAS-AB stain is the worst choice in *H. pylori* detection (in comparison with H&E and Giemsa stains) with 40% sensitivity and 67.65% specificity. However, this stain is best in the detection of intestinal metaplasia, since it detects all cases (even those with a small focal focus) and it produces no false positives. Although some authors recommend the routine use of IHC stains in detecting *H. pylori* (14, 35), the majority recommend their use only if necessary, as in cases of ambiguous H&E or Giemsa stain morphology because their use is time-consuming and expensive (13, 36, 37).

## Conclusion

The data from this study suggest a strong recommendation to use H&E, Giemsa and PAS-AB as a routine panel of stains in all gastric biopsies aiming for better viewing and interpretation of histopathologic morphology as well as for *H. pylori* detection. However, it is also recommended not to use PAS-AB stain for the detection of *H. pylori* infection.

## Figures and Tables

**Table 1:** Types of gastric mucosa, gastritis and gastritis types associated with *H. pylori* infection in this study

Types of gastric mucosa	No.	Types of gastritis in general	No.	Types of gastritis associated with *H. pylori* infection	No.
Antrum	21	Chronic active	15	Chronic active	13
Body	15	Chronic	34	Chronic	2
Antrum and body	13				

**Total**	**49**	**Total**	**49**	**Total**	**15**

**Table 2:** Uses of H&E and PAS-AB stains for the detection of intestinal metaplasia. Total actual number of IM cases is 3 out of 49 cases

Stain	No. of detected cases	Sensitivity (%)	Specificity (%)
IM cases detected by H&E stain	2	66.67	100
IM cases detected by PAS-AB stain	3	100	100

Note: IM biopsies were used as positive controls for PAS-AB stains

**Table 3:** Evaluation of better staining method for the detection of *H. pylori* in gastric biopsies

Stain	No. of positive cases	Sensitivity (%)	Specificity (%)
*H. pylori* cases detected by Giemsa stain	14	93.33	99.9
*H. pylori* cases detected by PAS-AB stain	17	40.00	67.65
*H. pylori* cases detected by H&E stain	13	66.67	91.18

Notes: Total actual number of *H. pylori* Infection is 15 out of 49 cases; Evaluation was done with 95% confidence interval.

Sensitivity**:** Geimsa versus PAS-AB, *P* = 0.02; Geimsa versus H&E, *P* = 0.046; PAS-AB versus H&E, *P* = 0.079

Specificity**:** Geimsa versus PAS-AB, *P* = 0.013; Geimsa versus H&E, *P* = 0.137; PAS-AB versus H&E, *P* = 0.046

## References

[1] Crowe SE (2019). *Helicobacter pylori* infection. N Engl J Med.

[2] Marshall BJ, Armstrong JA, McGechie DB, Glancy RJ (1985). Attempt to fulfil Koch’s postulates for pyloric *Campylobacter*. Med J Aust.

[3] Abadi ATB (2018). Diagnosis of *Helicobacter pylori* using invasive and noninvasive approaches. J Pathog.

[4] Smolka AJ, Schubert ML (2017). *Helicobacter pylori*-induced changes in gastric acid secretion and upper gastrointestinal disease. Curr Top Microbiol Immunol.

[5] McClain MS, Beckett AC, Cover TL (2017). *Helicobacter pylori* vacuolating toxin and gastric cancer. Toxins (Basel).

[6] Abadi ATB, Ierardi E, Lee YY (2015). Why do we still have *Helicobacter pylori* in our stomachs. Malays J Med Sci.

[7] Olbe L, Hamlet A, Dalenbäck J, Fändriks L (1996). A mechanism by which *Helicobacter pylori* infection of the antrum contributes to the development of duodenal ulcer. Gastroenterology.

[8] Malfertheiner P, Chan FK, McColl KE (2009). Peptic ulcer disease. Lancet.

[9] Ricci C, Holton J, Vaira D (2007). Diagnosis of *Helicobacter pylori*: invasive and non-invasive tests. Best Pract Res Clin Gastroenterol.

[10] Kassem E, Naamna M, Mawassy K, Beer-Davidson G, Muhsen K (2017). *Helicobacter pylori* infection, serum pepsinogens, and pediatric abdominal pain: a pilot study. Eur J Pediatr.

[11] Wong BC, Wong WM, Wang WH, Tang VS, Young J, Lai KC (2001). An evaluation of invasive and non-invasive tests for the diagnosis of *Helicobacter pylori* infection in Chinese. Aliment Pharmacol Ther.

[12] Cosgun Y, Yildirim A, Yucel M, Karakoc AE, Koca G, Gonultas A (2016). Evaluation of invasive and noninvasive methods for the diagnosis of *Helicobacter pylori* infection. Asian Pac J Cancer Prev.

[13] Patel SK, Pratap CB, Jain AK, Gulati AK, Nath G (2014). Diagnosis of *Helicobacter pylori*: what should be the gold standard?. World J Gastroenterol.

[14] Lash RH, Genta RM (2016). Routine anti-*Helicobacter* immunohistochemical staining is significantly superior to reflex staining protocols for the detection of Helicobacter in gastric biopsy specimens. Helicobacter.

[15] Pajares-García JM (1998). Diagnosis of *Helicobacter pylori*: invasive methods. Ital J Gastroenterol Hepatol.

[16] Kocsmár É, Szirtes I, Kramer Z, Szijártó A, Bene L, Buzás GM (2017). Sensitivity of *Helicobacter pylori* detection by Giemsa staining is poor in comparison with immunohistochemistry and fluorescent in situ hybridization and strongly depends on inflammatory activity. Helicobacter.

[17] Miftahussurur M, Yamaoka Y (2015). Appropriate first-line regimens to combat *Helicobacter pylori* antibiotic resistance: an Asian perspective. Molecules.

[18] Miftahussurur M, Shiota S, Suzuki R, Matsuda M, Uchida T, Kido Y (2015). Identification of *Helicobacter pylori* infection in symptomatic patients in Surabaya, Indonesia, using five diagnostic tests. Epidemiol Infect.

[19] Rimbara E, Sasatsu M, Graham DY (2013). PCR detection of *Helicobacter pylori* in clinical samples. Methods Mol Biol.

[20] Akeel M, Elmakki E, Shehata A, Elhafey A, Aboshouk T, Ageely H (2018). Prevalence and factors associated with *H. pylori* infection in Saudi patients with dyspepsia. Electron Physician.

[21] Salih BA (2009). *Helicobacter pylori* infection in developing countries: the burden for how long?. Saudi J Gastroenterol.

[22] Khakoo SI, Lobo AJ, Shepherd NA, Wilkinson SP (1994). Histological assessment of the Sydney classification of endoscopic gastritis. Gut.

[23] Makristathis A, Hirschl AM, Mégraud F, Bessède E (2019). Review: diagnosis of *Helicobacter pylori* infection. Helicobacter.

[24] Rudnicka K, Graczykowski M, Tenderenda M, Chmiela M (2014). *Helicobacter pylori* morphological forms and their potential role in the transmission of infection. Postepy Hig Med Dosw.

[25] Dominis M, Dzebro S, Gasparov S, Buljevac M, Colić-Cvrlje V, Banić M (2002). Morphology of gastritis and *Helicobacter pylori* infection. Lijec Vjesn.

[26] Stolte M, Meining A (2001). The updated Sydney system: classification and grading of gastritis as the basis of diagnosis and treatment. Can J Gastroenterol.

[27] Mawlood AH, Kawther RS, Balaky STJ (2019). Evaluation of invasive and non-invasive methods for the diagnosis of *H. pylori* in dyspepsia patients. Diyala Journal of Medicine.

[28] Batts KP, Ketover S, Kakar S, Krasinskas AM, Mitchell KA, Wilcox R (2013). Appropriate use of special stains for identifying *Helicobacter pylori*: recommendations from the Rodger C. Haggitt Gastrointestinal Pathology Society. Am J Surg Pathol.

[29] Wright CL, Kelly JK (2006). The use of routine special stains for upper gastrointestinal biopsies. Am J Surg Pathol.

[30] Fallone CA, Barkun AN, Loo VG, Lough J (2008). Hematoxylin and eosin staining of gastric tissue for the detection of *Helicobacter pylori*. Helicobacter.

[31] Panarelli NC, Ross DS, Bernheim OE, Landzgerg ZB, Schuetz AN, Jenkins SG (2015). Utility of ancillary stains for *Helicobacter pylori* in near-normal gastric biopsies. Hum Pathol.

[32] Pittman ME, Khararjian A, Wood LD, Montgomery EA, Voltaggio L (2016). Prospective identification of *Helicobacter pylori* in routine gastric biopsies without reflex ancillary stains is cost-efficient for our health care system. Hum Pathol.

[33] Laine L, Lewin DN, Naritoku W, Cohen H (1997). Prospective comparison of H&E, Giemsa and Genta stains for the diagnosis of *Helicobacter pylori*. Gastrointest Endosc.

[34] Rotimi O, Cairns A, Gray S, Moayyedi P, Dixon MF (2000). Histological identification of *Helicobacter pylori*: comparison of staining methods. J Clin Pathol.

[35] Jonkers D, Stobberingh E, de Bruine A, Arends JW, Stockbrügger R (1997). Evaluation of immunohistochemistry for the detection of *Helicobacter pylori* in gastric mucosal biopsies. J Infect.

[36] Ginestet F, Guibourg B, Doucet L, Théreaux J, Robaszkiewicz M, Marcorelles P (2017). Upfront immunohistochemistry improves specificity of *Helicobacter pylori* diagnosis: a French pathology laboratory point of view. Helicobacter.

[37] Tajalli R, Nobakht M, Mohammadi-Barzelighi H, Agah S, Rastegar-Lari A, Sadeghipour A (2013). The immunohistochemistry and toluidine blue roles for *Helicobacter pylori* detection in patients with gastritis. Iran Biomed J.

